# Comparison of category and letter fluency tasks through automated analysis

**DOI:** 10.3389/fpsyg.2023.1212793

**Published:** 2023-10-11

**Authors:** Carmen Gonzalez-Recober, Naomi Nevler, Sanjana Shellikeri, Katheryn A. Q. Cousins, Emma Rhodes, Mark Liberman, Murray Grossman, David Irwin, Sunghye Cho

**Affiliations:** ^1^Penn Frontotemporal Degeneration Center, University of Pennsylvania, Philadelphia, PA, United States; ^2^Linguistic Data Consortium, University of Pennsylvania, Philadelphia, PA, United States

**Keywords:** digital speech, verbal fluency task, category fluency task, letter fluency task, automated analysis

## Abstract

**Introduction:**

Category and letter fluency tasks are commonly used neuropsychological tasks to evaluate lexical retrieval.

**Methods:**

This study used validated automated methods, which allow for more expansive investigation, to analyze speech production of both category (“Animal”) and letter (“F”) fluency tasks produced by healthy participants (*n* = 36) on an online platform. Recordings were transcribed and analyzed through automated pipelines, which utilized natural language processing and automatic acoustic processing tools. Automated pipelines calculated overall performance scores, mean inter-word response time, and word start time; errors were excluded from analysis. Each word was rated for age of acquisition (AoA), ambiguity, concreteness, frequency, familiarity, word length, word duration, and phonetic and semantic distance from its previous word.

**Results:**

Participants produced significantly more words on the category fluency task relative to the letter fluency task (*p* < 0.001), which is in line with previous studies. Wilcoxon tests also showed tasks differed on several mean speech measures of words, and category fluency was associated with lower mean AoA (*p*<0.001), lower frequency (*p* < 0.001), lower semantic ambiguity (*p* < 0.001), lower semantic distance (*p* < 0.001), lower mean inter-word RT (*p* = 0.03), higher concreteness (*p* < 0.001), and higher familiarity (*p* = 0.02), compared to letter fluency. ANOVAs significant interactions for fluency task on total score and lexical measures showed that lower category fluency scores were significantly related to lower AoA and higher prevalence, and this was not observed for letter fluency scores. Finally, word-characteristics changed over time and significant interactions were noted between the tasks, including word familiarity (*p* = 0.019), semantic ambiguity (*p* = 0.002), semantic distance (*p*=0.001), and word duration (*p*<0.001).

**Discussion:**

These findings showed that certain lexical measures such as AoA, word familiarity, and semantic ambiguity were important for understanding how these tasks differ. Additionally, it found that acoustic measures such as inter-word RT and word duration are also imperative to analyze when comparing the two tasks. By implementing these automated techniques, which are reproducible and scalable, to analyze fluency tasks we were able to quickly detect these differences. In future clinical settings, we expect these methods to expand our knowledge on speech feature differences that impact not only total scores, but many other speech measures among clinical populations.

## 1. Introduction

Verbal fluency tasks, such as letter and category naming, are often used in neuropsychological testing to assess executive functioning and working memory of participants in both clinical and research settings (Elvevåg et al., 2002; Libon et al., 2009; Juhasz et al., 2012; Cook et al., 2014; Van Den Berg et al., 2017). The category fluency task, sometimes referred to as a semantic fluency task, directs participants to list as many words within a particular semantic category (i.e., “animals,” “vegetables,” “furniture”) as possible in a restricted time period (e.g., 1 min). The letter fluency task, known as a phonemic naming task, gives participants a letter (e.g., “f,” “s”) and asks that they list words that begin with that alphabetical character. Both variations of verbal fluency tasks have shown some common effects such as a correlation between reduced semantic fluency and overall fluency task performance (Libon et al., 2009). Many studies (e.g., Shao et al., 2014; Abdel Aziz et al., 2017; Vonk et al., 2019) have found that there are broad determinants that cause differences between category and letter fluency tasks, such as vocabulary knowledge, lexical access speed, demographics, and cortical thickness. While previous studies have compared these tasks, many studies have used manual counting methods, and manual scoring is still the primary method for fluency task analysis in clinical settings. While valid, this method leaves room for more human errors and does not allow for quick in-depth analysis of speech and lexical characteristics. Our study addresses this issue by showing how validated automated analysis processes can give a comprehensive look at the variability that exists within speech features between these tasks.

Digitizing the fluency tasks and applying advanced language technologies to automatically score them and to extract diverse additional speech features can maximize cognitive assessments, make them more accessible, and improve standardization for clinical research. Digitization and automatic processing allow researchers to measure informative features, such as inter-word response time (RT) and exact timestamps for the start and end of each word produced within a fluency task, which are not easily measured without digitized recordings and sophisticated speech technologies. These technologies also enable rapid automatic scoring of multiple features, such as lexical characteristics including word age of acquisition (AoA) and familiarity, which can lead to deeper characterization of an individual’s speech. Thus, automatic analyses of these word characteristics of fluency tasks may provide a quick, practical, easy-to-apply, and scalable tool that can be used in various settings, including clinical ones. By developing these tools in healthy speakers, they may be applied in the future to the clinical setting to characterize cognitive and language deficits, such as those seen in neurodegenerative disease and many other conditions.

Previous studies in healthy-controls and patients with neurodegeneration have compared performance between semantic and phonetic verbal fluency using manual processing methods. It is known that letter fluency task produces lower fluency scores (i.e., fewer valid words listed) than category fluency task (Shao et al., 2014). Some studies have looked at how this effect can be impacted by specific disorders, such as Attention-deficit/hyperactivity disorder (ADHD) and found that people with ADHD scored significantly lower in phonemic fluency task than controls, but the effect was not seen in semantic fluency task (Andreou and Trott, 2013). Verbal fluency tasks have also been found to be affected by psychosis such as schizophrenia. van Beilen et al. (2004) found that patients diagnosed with schizophrenia produced fewer words during the category fluency task than healthy controls, although they had similar clustering and switching patterns (i.e., naming words that fit into a sub-group and switching between sub-groups; Troyer et al., 1997) as healthy controls. Multiple studies have identified AoA as an important variable for prediction of fluency scores, as well as prediction of disease in patients (Monsch et al., 1992; Forbes-Mckay et al., 2005; Sailor et al., 2011). Familiarity of words produced might help differentiate primary progressive aphasia (PPA) diagnoses (Rofes et al., 2019), validating its importance in fluency task research. Sailor et al. (2011) found that tasks also differed by word frequency; frequency was higher on average in the letter fluency task, and also significantly higher in Alzheimer’s disease (AD) patients than healthy controls. In healthy older adults, Shao et al. (2014) found that lexical access speed and vocabulary knowledge had a larger effect on category fluency tasks than letter fluency tasks. Yet, there are limitations to manual studies which often constrained to probing a single aspect of fluency tasks. Indeed, Amunts et al. (2021) used a machine learning approach to test whether semantic verbal fluency tasks predicted executive functioning performance; they found that models were able to better predict the executive functioning task scores when using a full set of semantic verbal fluency features, including speech measures such as semantic distance than when using overall score of verbal fluency. This study highlighted the importance of using more in-depth lexical and speech measures when analyzing verbal fluency tasks, rather than only focusing on the sum of correct responses (Amunts et al., 2021).

Previous studies such as Troyer et al. (1997, 1998) and Raoux et al. (2008) have focused on the effects of clustering and cluster switching in these tasks, which provided valuable findings in not only healthy speakers but also patients with neurodegenerative condition. For example, Troyer et al. (1998) found that while overall fluency scores did not differ significantly between patients with AD and Parkinson’s disease (PD) and controls, the effects on switching and clustering of words did differ, where AD patients produced smaller clusters in both tasks than PD patients and healthy speakers. However, while manual analysis of clustering and switching is possible, as seen in the studies mentioned above, the grouping of words has varied depending on which clustering system studies followed and manual clustering of words was time consuming. For this reason, some previous studies have demonstrated that there are benefits to using automated methods to measure word clustering and switching effects (Pakhomov and Hemmy, 2014; Amunts et al., 2021; Nevado et al., 2021). With the automated computerized methods used by Pakhomov and Hemmy (2014), for example, they were able to implement a more objective approach to assessment of semantic clustering throughout the task and therefore create a more standardized and efficient approach than manual approaches to analyze other measures that can be indicative of neurodegeneration and noted through the verbal fluency tasks.

While possible to manually assess, rate, and compare words listed throughout these tasks, the ability to do this efficiently on large data sets becomes more feasible when using automated methods. A similar automated pipeline to the one used in this study was also used previously in various studies conducted on picture description tasks to collect these same novel measures that are unique to automated processes (Cho et al., 2021). In this comprehensive study, we use complete automated methods to simultaneously compare several linguistic features from letter and categorical fluency tasks. Our study builds on the work from Cho et al. (2021), which applied automated analysis to assess letter fluency, to further compare lexical and acoustic features of words produced throughout both category and letter fluency tasks in young, healthy participants. We compared “Animal” and “F” letter fluency tasks because they are the most common versions used in clinical research settings. We investigated how various lexical and speech features – including AoA, ambiguity, concreteness, frequency, familiarity, word length, phonetic and semantic distance, and word duration – correlated with overall fluency scores, how they changed over time throughout the task, and how these relationships differed by task.

Based on previous findings, we first hypothesized that overall fluency scores would be higher in the category fluency task than in the letter fluency task based on previous findings (e.g., Shao et al., 2014), and that the total scores of both tasks would be associated with language measures, such as higher AoA (e.g., Forbes-Mckay et al., 2005). Second, we hypothesized that tasks would differ in lexical characteristics driven by task requirements. Specifically, we expected task differences in the semantic and phonetic distance between two consecutive words, such as more phonetically distant words but less semantic distance in the category fluency task, and the converse pattern in the letter fluency task. Similarly, we expected that words produced during category fluency task would have lower mean semantic ambiguity, and therefore higher concreteness, because words fall within the same group (animal). We also expected to see lower mean AoA and higher mean familiarity in category fluency task because animals are taught during childhood and are identifiable by most. A lower mean frequency was also expected in category fluency task because animals are not used as commonly in everyday speech as some words beginning with “f” (e.g., *food, friend, family*). Finally, we hypothesized that speech measures would change throughout the task, and that these trajectories would significantly differ by task.

## 2. Methods

### 2.1. Participants

Thirty-eight undergraduate students (20 females, 18 males), who self-reported as healthy native speakers of English without hearing or speaking difficulties, were recruited to participate in this study at the University of Pennsylvania using a subject pool. Participants had similar ages and education levels in efforts to attain the least influence from non-language factors as it has been shown that both these demographic elements can affect fluency task performance (Kim et al., 2013). Ages of participants ranged from 18 to 23 years old, with the mean age of volunteers being 20.03 ± 1.17 years. Students received course credit for their class to participate in this online research. This study was conducted at the Linguistic Data Consortium with approval from the Institutional Review Board of the University of the Pennsylvania.

### 2.2. Data collection

The study included various neuropsychological tests that would be completed in sequence and would take up to 20 min to complete in full. An online experiment platform was created using PCIbex (Zehr and Schwarz, 2018) to digitally record multiple speech tasks. Among these tasks, were letter and category fluency tasks, including “F” and “Animal” prompts. The order of tasks, except the first and last story-recall tasks, were randomly presented to participants. Participants completed each task without skipping until notified that they were finished with the study.

Tasks were unproctored, and completed remotely on students’ electronic devices that were compatible with the PCIbex computerized program. To ensure sound quality, there was a pre-session recording to quality-check environment before the actual tasks, and participants were instructed to be in a quiet space with minimal background noise and no distractions for the duration of the study. During the verbal fluency task, students were instructed that they would have 60 s to list as many words as possible, which either started with a specific letter or fell within a specific category. They were given the defined category (“Animals”) or letter (“F”) prompt at the exact moment the timer started to avoid any ability to formulate a list of responses prior to commencing. Once the 60 s was complete, the recording was automatically stopped, and they moved on to the next task until the study was complete. Recordings and metadata were automatically uploaded into a secure Amazon Web Services bucket. Once concluded, the recordings and metadata were gathered by the Linguistic Data Consortium for further analysis and processing.

### 2.3. Data processing and measurements

Recordings were orthographically transcribed by trained annotators with a standard protocol of the University of Pennsylvania’s Linguistic Data Consortium and processed through a quality check by senior annotators. Transcripts and recordings were then automatically forced-aligned using a modified version of Penn Phonetics forced aligner (Jiahong and Liberman, 2008) and manually validated for accuracy using Audacity® software. When reviewing the alignment outputs, we checked that animals listed with two or more words (e.g., “polar bear,” “black bear,” “blue jay”) were counted as one expression and not mistaken for two unassociated words or multiple repetitions, leading to exclusion from the total score. In order to automatically process the data, we adopted the automated pipeline that calculated the number of correct responses and rated several lexical measures for each correct response for the letter fluency (Cho et al., 2021; available at https://github.com/csunghye/letter_fluency_pipeline) and built a similar automated pipeline for the category fluency (available at https://github.com/csunghye/category_fluency). For letter fluency, the pipeline tagged all words for their part-of-speech categories using spaCy (Honnibal and Johnson, 2015), and counted all words starting with “f,” but excluding repetitions, proper nouns (e.g., “Fanta,” PROPN in spaCy), and numbers (e.g., “five,” NUM in spacy) from the participant’s total score. For category fluency, WordNet (Fellbaum, 2005), a lexical database which offers semantic relationships between words within specific categories, including animals, was used from the textblob package (Loria, 2018) in Python to automatically count the number of correct responses (hyponyms of “animal”) of the animal fluency task. Phrases which did not include animals or F-letter words (e.g., “I cannot think of anything else”) were excluded in scoring and in automated analysis of speech features within the tasks.

Our pipelines automatically measured acoustic features and tagged words for various lexical measures over the time course of each fluency task. Forced-alignment timestamps marked the word start time and the word end time. Two acoustic measures were calculated: (1) word duration (seconds) was calculated by the difference between the beginning and ending of a word, and (2) inter-word RT, calculated as the difference between word start time and its antecedent’s end time. RTs were calculated between valid words, so fillers (e.g., “hmm” or “um”) and words that did not fall into the given category were omitted.

Using published norms, the algorithm automatically rated each valid word for the number of phonemes and 5 lexical measures: AoA as the average age a word is acquired (Brysbaert et al., 2018), semantic ambiguity as the number of different meanings which a word has in a context (Hoffman et al., 2013), frequency as count per million words in a large-scale corpus (Brysbaert and New, 2009), familiarity as the z-scored percentage of people who know what a given word is Brysbaert et al. (2018), and semantic concreteness as speakers’ judgment on how concrete a word referent is on a scale of one to five (Brysbaert et al., 2014). Further, semantic and phonetic distances were calculated between each pair of consecutive valid words by comparing each word to its antecedent. Semantic distance – how closely related two words are by meaning – was measured as the Euclidean distance between the 300-dimension word vector representations from GloVe (Pennington et al., 2014). Phonetic distance – the distance between pronunciation of two successive words – was measured by calculating the time-warped normalized Euclidean distance between 13 Mel-frequency Cepstrum Coefficients of the two consecutive words (Cho et al., 2021).

### 2.4. Statistical analysis

First, category and letter fluency task total scores were tested for normality using Shapiro–Wilk tests. Wilcoxon test compared total scores between the two fluency tasks since the category fluency scores were not normally distributed. We reported Spearman’s correlation between the total scores for the two fluency tasks. We tested the relation between total fluency score (dependent variable) and each averaged speech measure for each fluency task using linear regression models (total: *n* = 2 acoustic measures, *n* = 8 lexical-semantic measures). We tested if the relationship between averaged speech measures and total fluency scores differed by task (letter vs. category) using interactions (total score ~ mean lexical measure*task). ANOVAs compared the interaction models with the main-effects only models.

We compared the averaged speech measures between the two fluency tasks using paired Wilcoxon tests (mean speech measure ~ task).

To compare change in acoustic and lexical measures over the course of the fluency task (i.e., task time in seconds from recording onset), we performed time series analysis using linear mixed-effects interaction models that tested lexical measure (dependent variable) over task time (in seconds) by task (lexical measure ~ task time*task + (1|subject)).

To assess which speech features were most impactful in determining performance in each fluency task (i.e., total score), we ran backward selection linear regression models with the fluency score in each task being used as the dependent variable, while all averaged lexical-semantic measures (*n* = 8) were used as the independent variables. We reported the results of the best fit model for each fluency task. We used RStudio (RStudio Team, 2022) version 1.4.1717 to perform all statistical analysis in this study.

## 3. Results

Results below describe the relevant findings of the comparative analysis conducted between both tasks. Relationships between total fluency scores of each task (Section 3.1), total scores and speech measures by task (Section 3.2), speech measures between tasks (Section 3.3), and speech measure changes over time by task (Section 3.4) were analyzed and described below.

### 3.1. Total score comparison between fluency tasks

**Figure 1:**
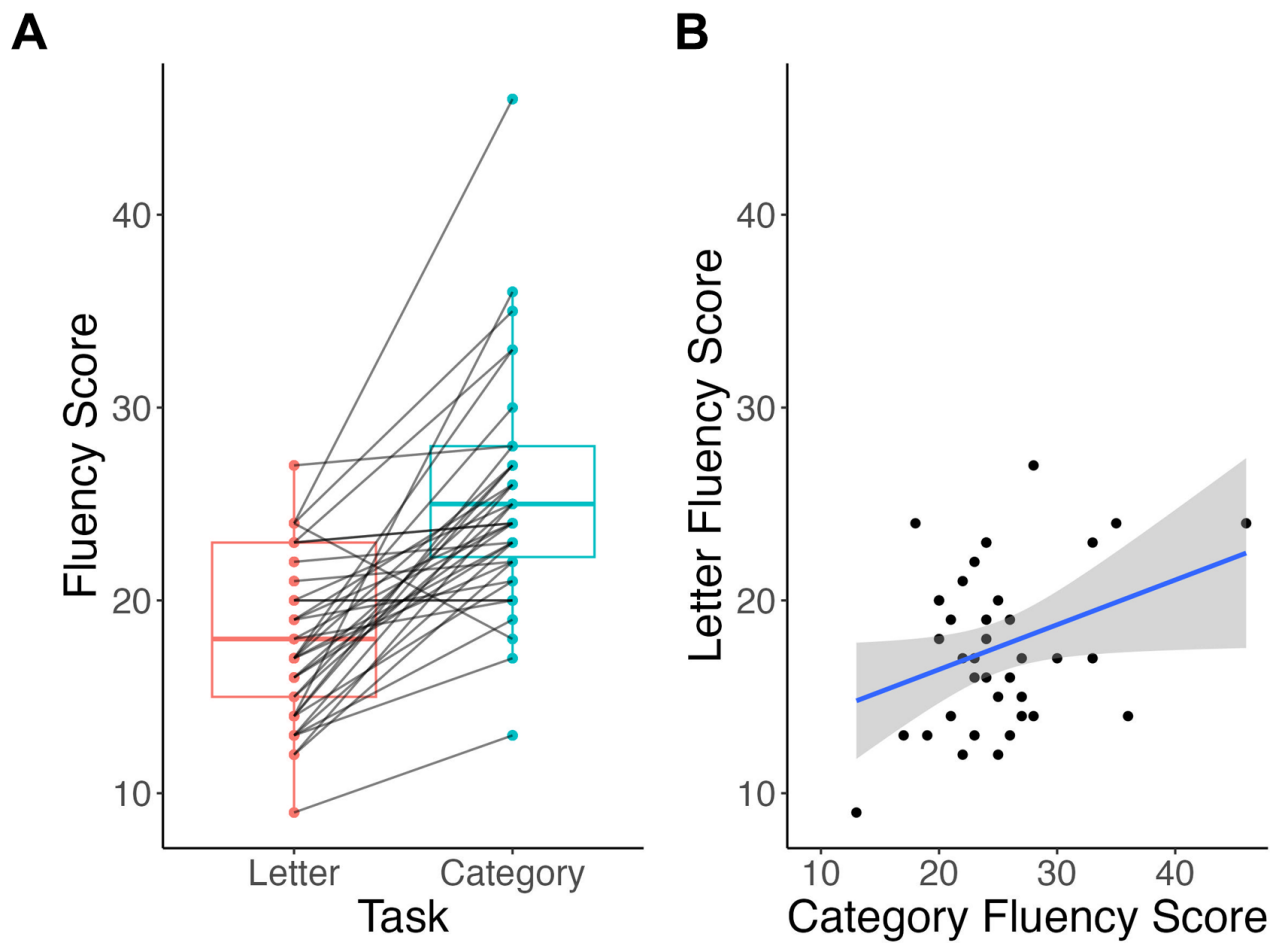
Within-subject differences **(A)** and correlation **(B)** between total scores of letter vs. category fluency tasks.

Participants in this study produced significantly higher scores on 1-min timed category fluency task than letter fluency tasks (V = 652, *p* < 0.001), when correct responses were counted automatically (Figure 1). The mean total score in the category naming (“Animal”) task was 26.35 ± 5.92 total words, while in the letter fluency (“F”) task it was 18.54 ± 4.19 words. Only 1 participant (of 38; 2.6%) scored higher in the letter fluency, and 2 participants (5.3%) had equivalent scores in both tasks. Total scores in letter and category fluency tasks were positively correlated (*t* = 2, *r* = 0.33, *p* = 0.04).

### 3.2. Relation between total fluency scores and speech measures by task

**Figure 2:**
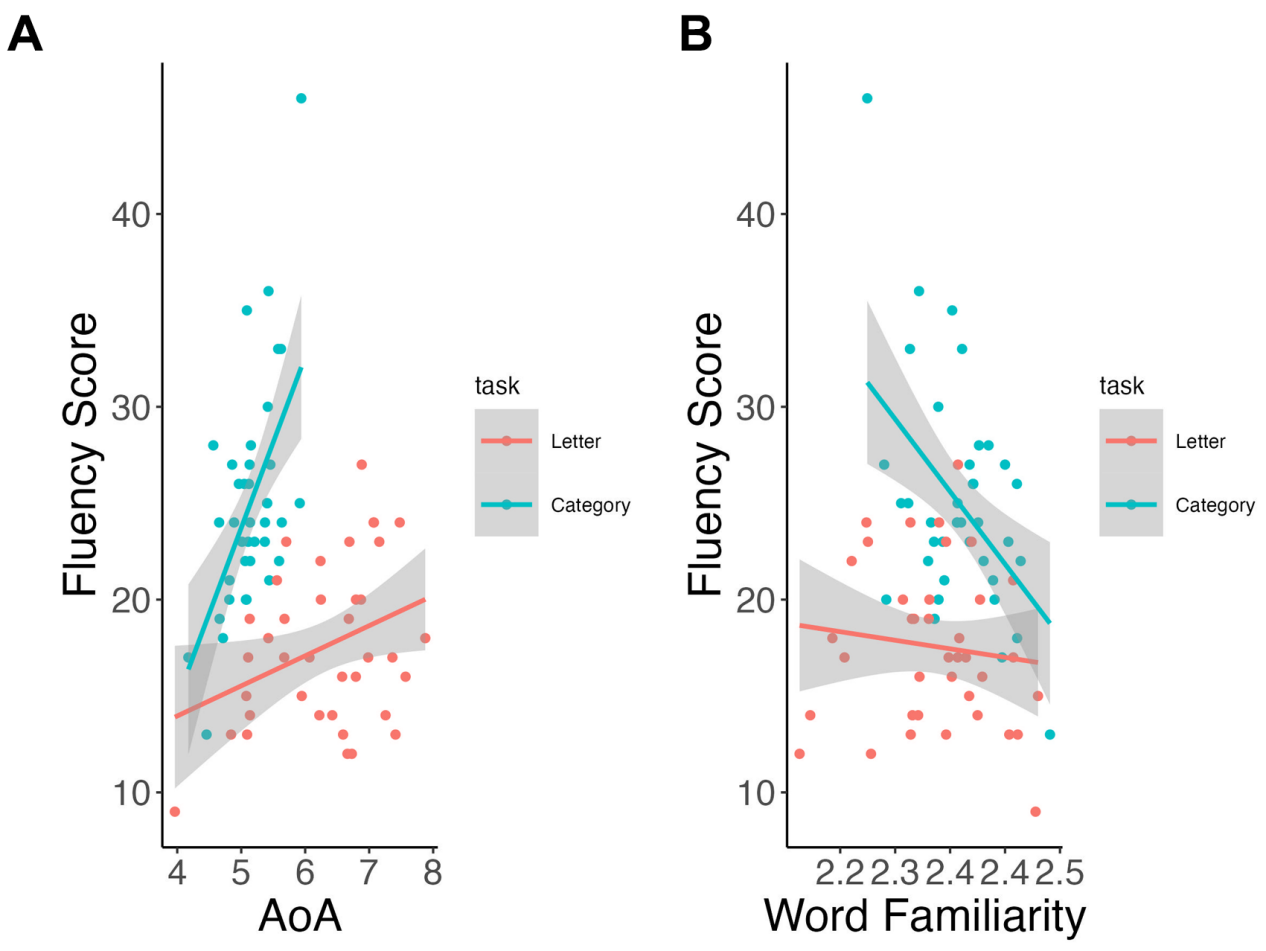
Relationship of lexical measures to fluency total scores by task. AoA **(A)**, word familiarity **(B)**.

Mean AoA and word familiarity showed significant interactions for task on total fluency scores (*p* < 0.001 and *p* = 0.004, respectively). Participants who produced later acquired words on average (i.e., higher mean AoA, β = 8.92, *p* < 0.001; Figure 2A) and less familiar words (β = −71.0, *p* < 0.001; Figure 2B) scored significantly higher only on the category fluency task (vs. letter fluency, *p* > 0.13). None of the other speech measures showed significant relation with total scores between or across tasks.

### 3.3. Differences in speech measures between tasks

**Figure 3:**
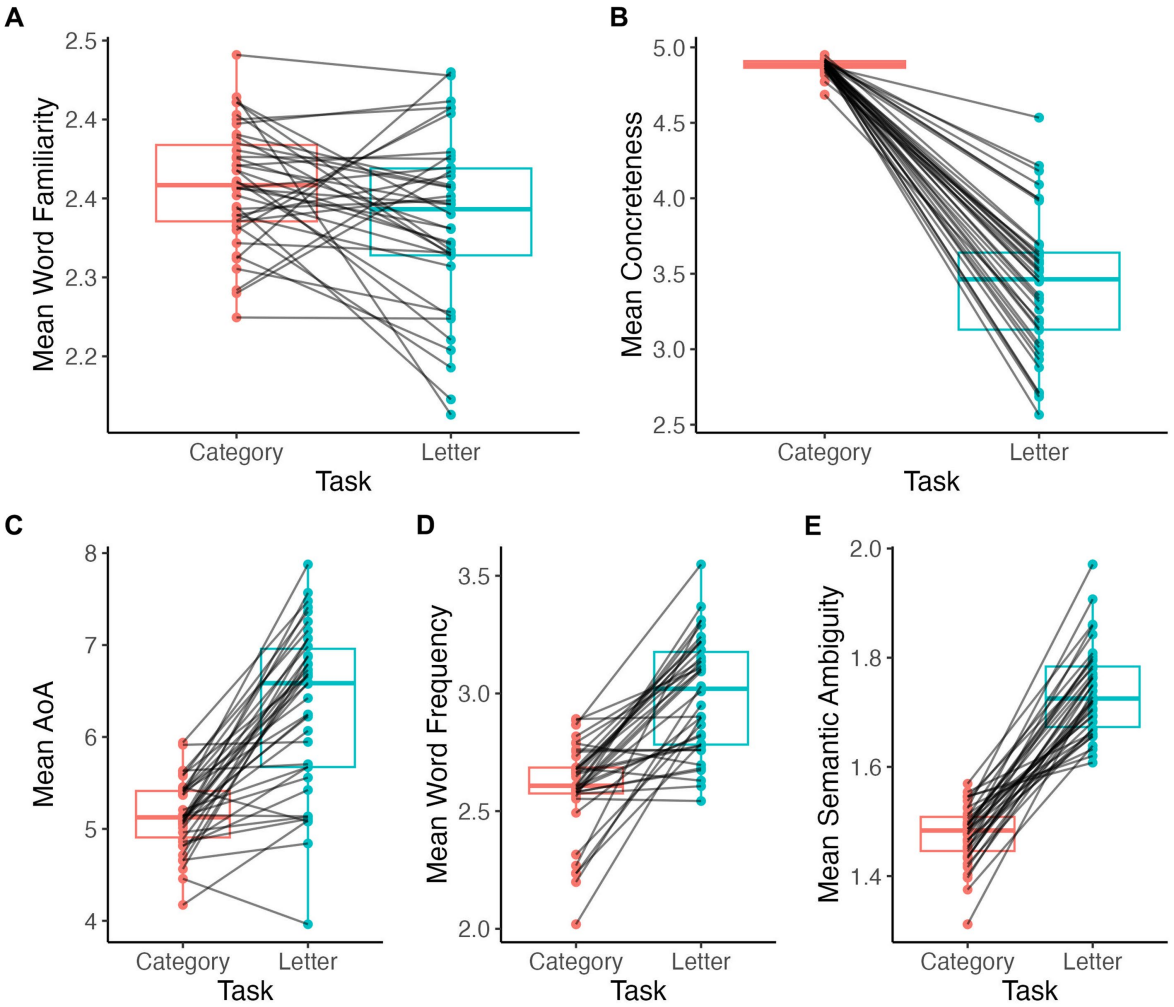
Comparison of mean lexical measures by task. Word familiarity **(A)**, concreteness **(B)**, AoA **(C)**, word frequency **(D)**, semantic ambiguity **(E)**.

Words produced during the category fluency task were significantly more familiar (V = 536, *p* = 0.02; Figure 3A) and more concrete (V = 741, *p* < 0.001; Figure 3B) than those produced during the letter fluency task. Also, words during the category fluency task were earlier acquired (i.e., lower AoA) (V = 18, *p* < 0.001; Figure 3C), less frequent (V = 17, *p* < 0.001; Figure 3D), and less ambiguous (V = 0, *p* < 0.001; Figure 3E) on average compared to the words produced during the letter fluency task.

**Figure 4:**
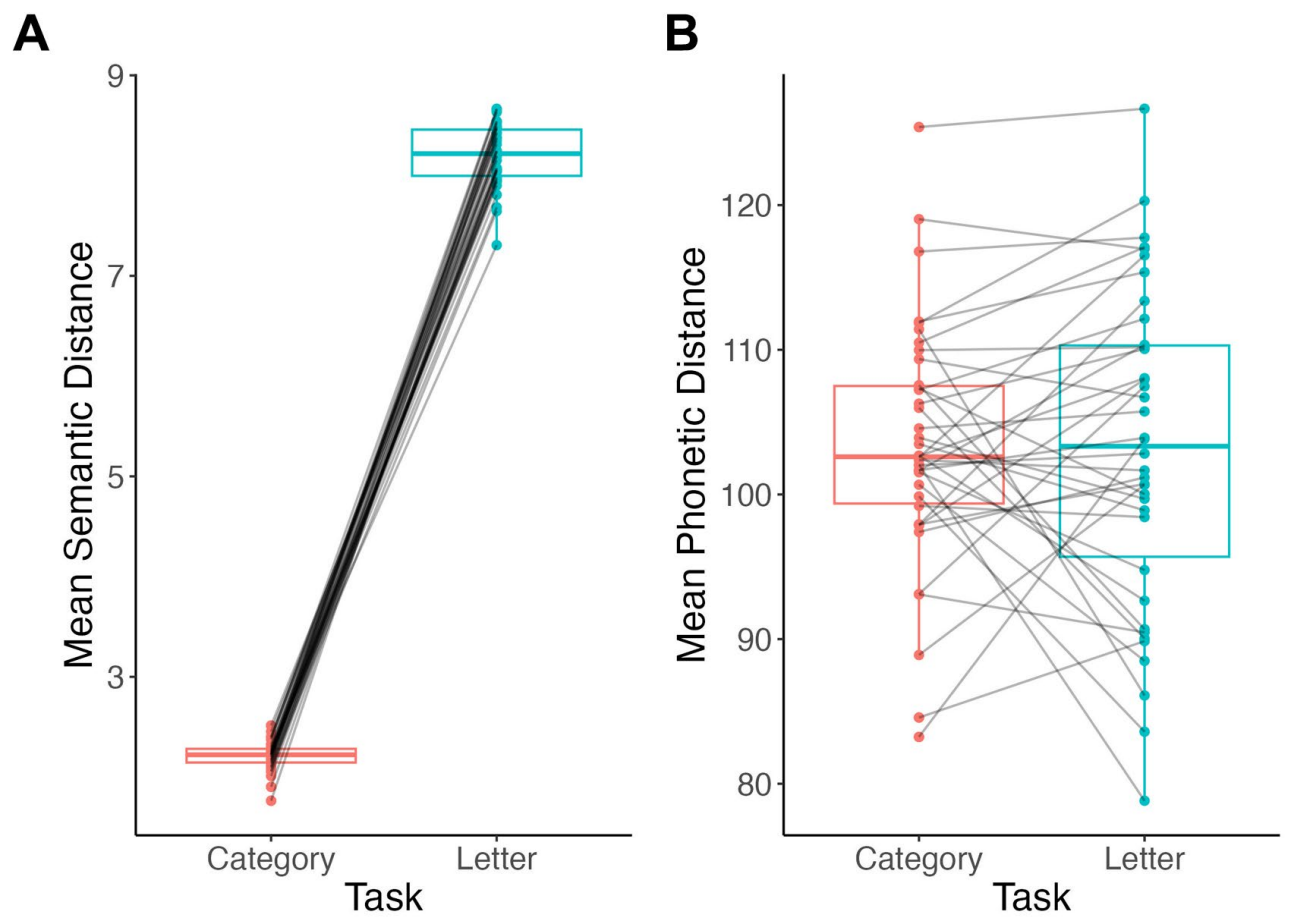
Comparison of lexical-semantic distance measures by task. Semantic distance **(A)**, phonetic distance **(B)**.

Participants produced significantly more semantically similar words in sequence during category fluency than during letter fluency tasks (V = 0, *p* < 0.001; Figure 4A). By contrast, mean phonetic distances did not differ between category and letter fluency tasks (V = 344, *p* = 0.7; Figure 4B).

**Figure 5:**
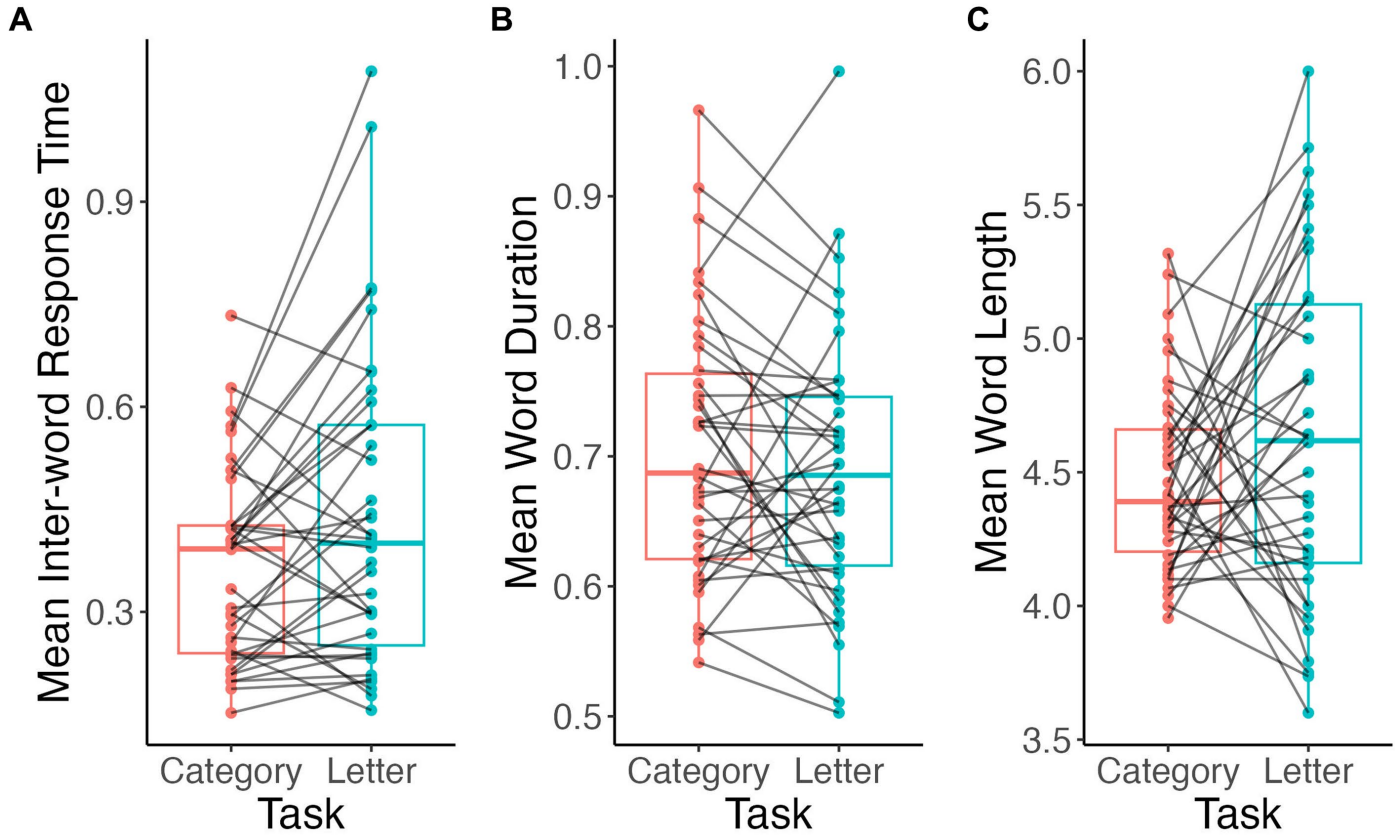
Comparison of acoustic and speech measures by task. Inter-word RT **(A)**, word duration **(B)**, word length **(C)**.

Mean inter-word RT were longer in letter fluency than in animal fluency (V = 222, *p* = 0.03; Figure 5A). Alternatively, mean word duration in seconds (V = 453, *p* = 0.2; Figure 5B) and word length in number of phonemes (V = 264, *p* = 0.2; Figure 5C) did not differ between tasks.

### 3.4. Time-series analysis: differences in change in speech measures over time by task

**Figure 6:**
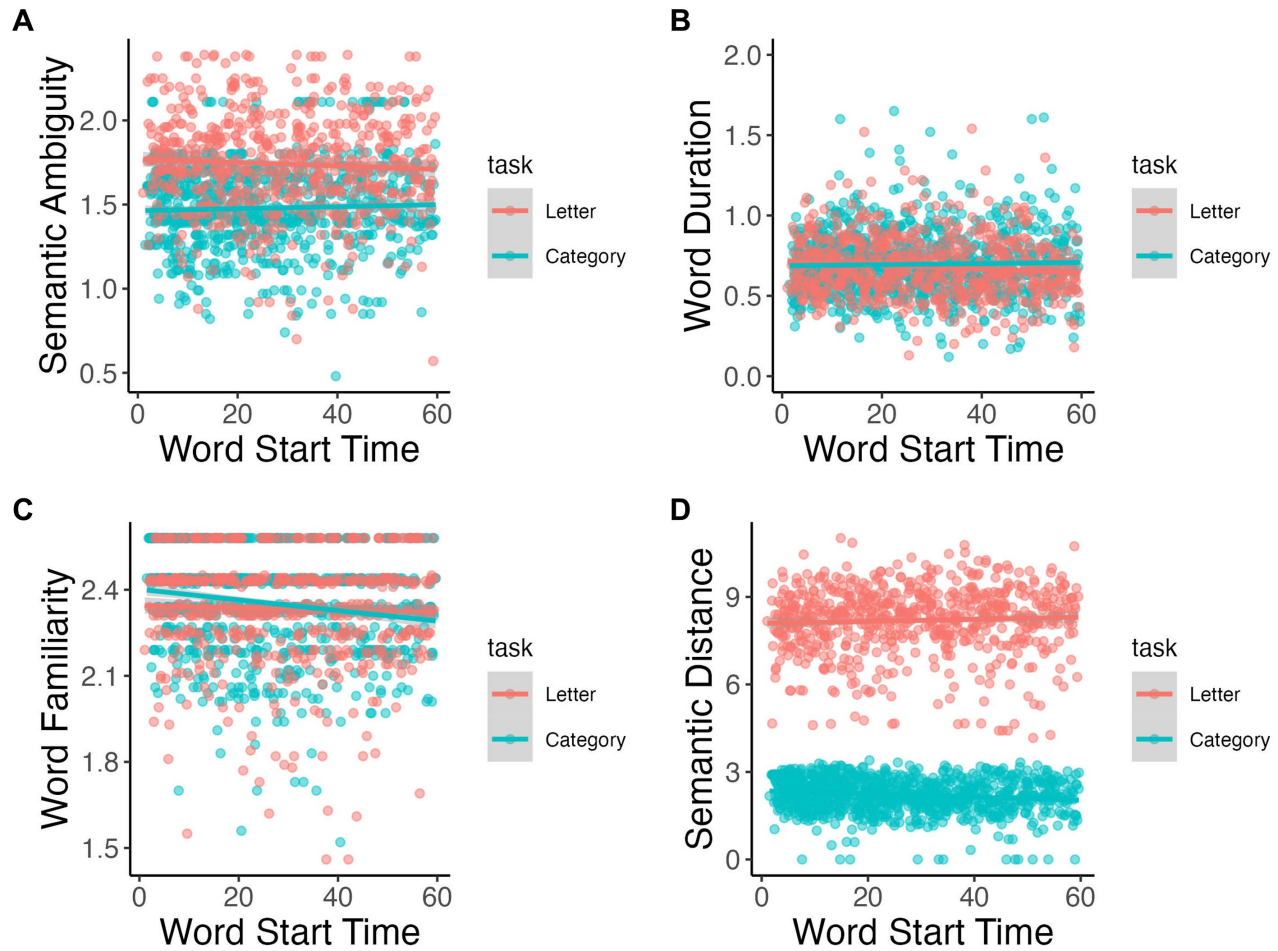
Semantic ambiguity **(A)**, word duration **(B)**, word familiarity **(C)**, and semantic distance **(D)** throughout task.

Change in semantic ambiguity of consecutive words significantly differed over time between tasks (*p* = 0.002; Figure 6A); however neither slope was statistically significant (*p* > 0.10). Word duration significantly interacted over time by task (*p* < 0.001; Figure 6B); word duration lengthened over time for category fluency task (β < 0.001, *p* = 0.032) and decreased over time for letter fluency task (β = −0.001, *p* < 0.001). Word familiarity and semantic distance also showed significant interactions with time by task (*p* = 0.019 and *p* = 0.001, respectively; Figures 6C,D): both lexical measures decrease over time only for category fluency task (familiarity: β = −0.002, *p* < 0.001; semantic distance: β = −0.005, *p* = 0.001).

### 3.5. Speech measures effect on total fluency scores among tasks

Our best-fit model for the animal category fluency task (adjusted *r*^2^ = 0.4181) showed that AoA (p < 0.001) and word duration (*p* = 0.023) were both significant in explaining animal fluency scores (Table 1). Alternatively, the best-fit model created for the “f” letter task (adjusted *r*^2^ = 0.3837) showed that AoA (*p* < 0.001) and word length in number of phonemes (*p* = 0.001) were significantly related with score prediction of the task (Table 2).

**Table 1:** The output of animal fluency task backward selection linear regression model.

	Estimate	Std. Error	*t* value	Pr (>|t|)
Intercept	−149.2	92.9	−1.6	0.118197
AoA	10.8	2.2	5	0.000022
Semantic ambiguity	26.1	14.5	1.8	0.081789
Semantic distance	−9.9	5.8	−1.7	0.094258
Concretness	23.5	17.7	1.3	0.192323
Word duration	−18	7.6	−2.4	0.023191

**Table 2:** The output of letter fluency task backward selection linear regression model.

	Estimate	Std. Error	*t* value	Pr (>|t|)
Intercept	−112.52	47.67	−2.4	0.0252
AoA	6.28	1.397	4.5	0.0001
Phonemes	−4.65	1.296	−3.6	0.0012
Familiarity	25.85	13.247	2	0.0607
Semantic ambiguity	12.57	9.383	1.3	0.1909
Phonetic distance	0.11	0.055	2	0.0604
Concreteness	4.14	2.584	1.6	0.1198
Inter-word RT	−3.5	2.767	−1.3	0.216
Word duration	8.86	6.124	1.4	0.1587

## 4. Discussion

Category and letter fluency tasks are often used throughout research and clinical practice to assess working memory, executive functioning, semantic functioning, and verbal functioning, specifically lexical access ability (Shao et al., 2014). While common practice has been to manually score these tasks, in this study, we used automated methods to test the relationships between varying language characteristics, how they were related to overall fluency scores, and how they changed over time in both category fluency and letter fluency tasks. Our automated results of overall fluency score comparison are in line with previous studies that used manual counting (e.g., Sauzéon et al., 2011), and showed that overall scores in both tasks significantly differed, as described in Shao et al. (2014). As many previous studies suggest (Luo et al., 2010; Shao et al., 2014; Cho et al., 2021), the finding that participants tend to score higher on category fluency tasks than letter fluency tasks may be due to the conscious effort it takes for participants to suppress the impulse to list semantically related words during the letter fluency task, as semantic grouping is used more frequently in real-world situations. We also found that fluency scores in the category fluency task positively correlated with AoA (Forbes-Mckay et al., 2005) and negatively with word familiarity. We showed that several lexical characteristics, mean inter-word RT, and semantic distance between two consecutive words significantly differed depending on fluency task type. Our results also showed that word familiarity, semantic ambiguity, semantic distance, and duration of words produced during the tasks significantly changed over time. When lexical variables were assessed for their influence on the fluency score in each task, AoA was a significant factor in predicting both tasks’ overall scores, while word duration was important in predicting category fluency scores, and word length significantly explained letter fluency scores.

Our findings, which showed the importance of AoA in explaining overall fluency task scores, are in line with previous studies that demonstrate that AoA has a considerable effect on verbal fluency task scores on both semantic and phonetic fluency tasks. Forbes-McKay et al. (2005) showed that among AoA, typicality, and number of words produced, AoA was most valuable in predicting whether a person belonged to an elderly healthy participant or AD group. They found that although AoA did not work alone in identifying patient phenotype, a lower mean AoA score was useful for identification of controls versus AD patients. Sailor et al. (2011) also found that semantic fluency tasks had lower mean AoA than letter fluency tasks in both AD population and elderly healthy controls. Furthermore, they also saw that this effect was greater on AD patients than the controls and AD patients produced words that had lower mean AoA across both tasks. Monsch et al. (1992) also found significant impairment in AD patients when comparing them to healthy age-matched participants and found the most sensitivity of AoA in category fluency task. In our study with young healthy controls, we found a positive correlation between AoA and total score as participants with higher fluency scores produced words that were acquired later on average in both tasks, although only significant in the category fluency task, and there was a significant difference in mean AoA between tasks, as expected.

We also found unexpected results about phonetic and semantic distance between tasks. We hypothesized that word produced during the category fluency task would have lower semantic distance (i.e., more similar in meaning) and word produced during the letter fluency task would show lower phonetic distance (i.e., more similar in pronunciation), as category naming focuses on semantic grouping of words while letter fluency task focuses on phonetic grouping. As expected, we found significantly lower mean semantic distance between words in the category fluency task than we did in the letter fluency task. However, unlike results from previous studies, we did not find a significant difference for mean phonetic distance between tasks, and words produced during the letter fluency were not more phonetically similar than those produced during the category fluency task. A possible explanation for this finding is that participants would typically begin listing different animal species within a genus and repeat the same word within different responses listed (e.g., “black bear,” “polar bear,” “brown bear”). This phonetic similarity between words may have contributed to lower phonetic distance in category fluency task and therefore be the reason we did not see significant differences between the category and letter fluency tasks. As seen in previous studies, such as Cho et al. (2021), we observed that semantic distance increased over time in the letter fluency task, while we saw the opposite trend in category fluency task. This could also be attributed to the listing of similar animals mentioned above, as these tended to occur later in the task. These findings can also be related back to switching effects during letter and category fluency tasks as seen in studies such as Haugrud et al. (2011) which found that clustering and switching scores did not contribute relevant findings in the letter fluency task, while showing in the category fluency task that healthy controls switched more and produced more subcategories than AD patients.

Mean inter-word RT, which we were able to efficiently calculate through our automated pipeline, had a significant effect on total scores in both the category and letter tasks. This was in line with previous studies that showed significant effects in letter fluency task (Rohrer et al., 1995; Shao et al., 2014; Cho et al., 2021). Inter-word RT was longer, on average, in the letter fluency task compared to the category fluency task, which may explain in part the difference in total scores (Luo et al., 2010). Word duration became shorter over time in the letter fluency task, while increasing over time in the category fluency task. This could be attributed to the tendency to begin listing sub-categories of animals as the task goes on, causing longer words to be produced.

Studies, such as Rofes et al. (2019), have highlighted the importance of word familiarity in verbal fluency tasks specifically. They found that familiarity was one of the most important variables in distinguishing patients with svPPA (i.e., semantic variant PPA) and lvPPA (i.e., logopenic variant PPA). In our study, word familiarity significantly differed between tasks, and total scores were also significantly negatively related with mean familiarity in both tasks; however, this effect was greater in the category fluency task. One possible explanation for this difference might be that many animals that are frequently listed, such as farm animals or domestic animals, had relatively high familiarity scores, so for participants to score significantly higher, they must also produce less familiar animals. Participants also had higher total scores (i.e., producing more words) in the category fluency task, so they might have come up with more words that were less familiar. Even so, mean word familiarity was higher in category than in letter fluency task. This can be attributed to the general familiarity of animals among the population, while more uncommon words can be listed in the letter fluency task. Along with this, there were interactions between the tasks word familiarity over time as category fluency significantly decreased throughout the task. We also note that mean word frequency was significantly higher in the letter task, likely due to the fact that animal names are not words that are commonly mentioned outside of specific contexts and therefore not used as frequently.

This study showed that valid automated methods allow for comprehensive comparison of both category and letter fluency tasks. Although our results were in line with previous studies about these tasks, there were some limitations that could be improved upon in future research. We only examined young speakers with homogeneous education background, so the results might not generalize to speakers with different demographic characteristics. Participants self-reported no cognitive deficits and further testing was not completed, so results could not be contrasted to other cognitive exams. Also, conducting this study on patients with varying neurodegenerative disorders, or other populations, could help with further understanding of how these tasks differ in results between specific diseases. Although manual transcription is standardized and quality checked by trained annotators, applying automatic speech recognition (ASR) tools to fluency tasks may help reduce human efforts and costs. We have not explored this possibility yet, since most available ASR tools are trained with natural or read speech where words are connected to form sentences. These systems would perform poorly on fluency tasks, where produced words form a list of words, not natural sentences. Fine-tuning existing ASR systems in the future might help automatically transcribing fluency task outputs.

Overall, this study demonstrated the differences in word characteristics that were produced during category and letter fluency tasks in the same group of healthy individuals using fully automated methods. By using these methods to analyze both tasks, we were able to show that speech features of words listed, which can only be calculated through automated processing, can have implications on overall scores, which gives insight to unique differences between both tasks that cannot be easily recognized through manual analysis. Our study demonstrated that using automated speech and language processing technologies is possible and practical to analyze these tasks in large quantities of participants, enabling large-scale studies of patients with neurodegenerative disease in the future. By improving standardization of these tasks in clinical settings, we will be able to discover more unique speech features which could indicate further neurological impairment, such as deterioration of executive functioning, and will help explain patient performance throughout other varying neurological exams. Future work can apply these methods to patient data to provide a better understanding of precisely which differences could exist within the population, and eventually generate further knowledge of overall disease progression.

## Data availability statement

The raw data supporting the conclusions of this article will be made available by the authors upon request.

## Ethics statement

The studies involving humans were approved by Institutional Review Board of the University of Pennsylvania. The studies were conducted in accordance with the local legislation and institutional requirements. The participants provided their written informed consent to participate in this study.

## Author contributions

CG-R, SC, MG, and NN developed the research concept and design of this study. SC and ML collected the data as part of a larger scale project. CG-R and SC analyzed the data and attained the results and drafted the manuscript. CG-R, NN, SS, KC, ER, ML, MG, DI, and SC worked together on the interpretation of the results. SS, KC, and ER assisted in revision of the paper. All authors contributed to the article and approved the submitted version.

## Funding

This research was supported by the National Institutes of Health (K23AG083124-01, P01-AG-066597, AG073510-01), the Alzheimer’s Association (AARF-21-851126, AARF-D-619473), Department of Defense (W81XWH-20-1-0531), and American Academy of Neurology (#2022-2784).

## Conflict of interest

The authors declare that the research was conducted in the absence of any commercial or financial relationships that could be construed as a potential conflict of interest.

## Publisher’s note

All claims expressed in this article are solely those of the authors and do not necessarily represent those of their affiliated organizations, or those of the publisher, the editors and the reviewers. Any product that may be evaluated in this article, or claim that may be made by its manufacturer, is not guaranteed or endorsed by the publisher.

## References

[ref1] Abdel Aziz K. Khater M. S. Emara T. Tawfik H. M. Rasheedy D. Mohammedin A. S.. (2017). Effects of age, education, and gender on verbal fluency in healthy adult Arabic-speakers in Egypt. Appl. Neuropsychol. Adult 24, 331–341. doi: 10.1080/23279095.2016.1185424, PMID: 27282630

[ref2] Amunts J. Camilleri J. A. Eickhoff S. B. Patil K. R. Heim S. von Polier G. G.. (2021). Comprehensive verbal fluency features predict executive function performance. Sci. Rep. 11:6929. doi: 10.1038/s41598-021-85981-1, PMID: , 33767208 PMC7994566

[ref3] Andreou G. Trott K. (2013). Verbal fluency in adults diagnosed with attention-deficit hyperactivity disorder (ADHD) in childhood. Atten. Defic. Hyperact. Disord. 5, 343–351. doi: 10.1007/s12402-013-0112-z, PMID: , 23749309

[ref4] Brysbaert M. Mandera P. Keuleers E. (2018). Word prevalence norms for 62,000 English lemmas. Behav. Res. Methods 2018, 467–479. doi: 10.3758/s13428-018-1077-9 29967979

[ref5] Brysbaert M. New B. (2009). Moving beyond Kučera and Francis: A critical evaluation of current word frequency norms and the introduction of a new and improved word frequency measure for American English. Behav. Res. Methods 41, 977–990. doi: 10.3758/BRM.41.4.977 19897807

[ref6] Brysbaert M. Warriner A. B. Kuperman V. (2014). Concreteness ratings for 40 thousand generally known English word lemmas. Behav. Res. Methods 46, 904–911. doi: 10.3758/s13428-013-0403-5, PMID: 24142837

[ref7] Cho S. Nevler N. Parjane N. Cieri C. Liberman M. Grossman M.. (2021). Automated analysis of digitized letter fluency data. Front. Psychol. 12:654214. doi: 10.3389/fpsyg.2021.654214, PMID: 34393894 PMC8359864

[ref8] Cho S. Nevler N. Shellikeri S. Parjane N. Irwin D. J. Ryant N.. (2021). Lexical and acoustic characteristics of young and older healthy adults. J. Speech Lang. Hear. Res. 64, 302–314. doi: 10.1044/2020_JSLHR-19-00384, PMID: 33439761 PMC8632482

[ref9] Cook P. A. McMillan C. T. Avants B. B. Peelle J. E. Gee J. C. Grossman M. (2014). Relating brain anatomy and cognitive ability using a multivariate multimodal framework. NeuroImage 99, 477–486. doi: 10.1016/j.neuroimage.2014.05.008, PMID: 24830834 PMC4151353

[ref10] Elvevåg B. Fisher J. E. Gurd J. M. Goldberg T. E. (2002). Semantic clustering in verbal fluency: Schizophrenic patients versus control participants. Psychol. Med. 32, 909–917. doi: 10.1017/S0033291702005597, PMID: 12171385

[ref11] Fellbaum C. (2005). “WordNet and wordnets,” in Encyclopedia of Language and Linguistics, *Second Edition*. ed. Keith B. (Oxford: Elsevier), 665–670.

[ref12] Forbes-Mckay K. E. Ellis A. W. Shanks M. F. Venneri A. (2005). The age of acquisition of words produced in a semantic fluency task can reliably differentiate normal from pathological age related cognitive decline. Neuropsychologia 43, 1625–1632. doi: 10.1016/j.neuropsychologia.2005.01.008, PMID: 16009244

[ref13] Haugrud N. Crossley M. Vrbanic M. (2011). Clustering and Switching Strategies During Verbal Fluency Performance Differentiate Alzheimer’s Disease and Healthy Aging. J. Int. Neuropsychol. Soc. 17, 1153–1157. doi: 10.1017/S1355617711001196, PMID: 22014065

[ref14] Hoffman P. Lambon Ralph M. A. Rogers T. T. (2013). Semantic diversity: A measure of semantic ambiguity based on variability in the contextual usage of words. Behav. Res. Methods 45, 718–730. doi: 10.3758/s13428-012-0278-x, PMID: 23239067

[ref15] Honnibal M. Johnson M. (2015). An improved non-monotonic transition system for dependency parsing. EMNLP 2015: Conference on empirical methods in natural language processing, 1373–1378.

[ref16] Jiahong Y. Liberman M. (2008). Speaker identification on the SCOTUS corpus. J. Acoust. Soc. Am. 123:3878. doi: 10.1121/1.2935783

[ref17] Juhasz B. J. Chambers D. Shesler L. W. Haber A. Kurtz M. M. (2012). Evaluating lexical characteristics of verbal fluency output in schizophrenia. Psychiatry Res. 200, 177–183. doi: 10.1016/j.psychres.2012.06.035, PMID: 22809852 PMC3513518

[ref18] Kim B. J. Lee C. S. Oh B. H. Hong C. H. Lee K. S. Son S. J.. (2013). A normative study of lexical verbal fluency in an educationally-diverse elderly population. Psychiatry Investig. 10, 346–351. doi: 10.4306/pi.2013.10.4.346, PMID: 24474982 PMC3902151

[ref19] Libon D. J. McMillan C. Gunawardena D. Powers C. Massimo L. Khan A.. (2009). Neurocognitive contributions to verbal fluency deficits in frontotemporal lobar degeneration. Neurology 73, 535–542. doi: 10.1212/WNL.0b013e3181b2a4f5, PMID: 19687454 PMC2730797

[ref9002] Loria S. (2018). textblob Documentation. Release 0.15, 2.

[ref20] Luo L. Luk G. Bialystok E. (2010). Effect of language proficiency and executive control on verbal fluency performance in bilinguals. Cognition 114, 29–41. doi: 10.1016/j.cognition.2009.08.014, PMID: 19793584

[ref21] Monsch A. U. Bondi M. W. Butters N. Salmon D. P. Katzman R. Thal L. J. (1992). Comparisons of verbal fluency tasks in the detection of dementia of the Alzheimer type. Arch. Neurol. 49, 1253–1258. doi: 10.1001/archneur.1992.00530360051017, PMID: , 1449404

[ref22] Nevado A. Del Río D. Martín-Aragoneses M. T. Prados J. M. López-Higes R. (2021). Preserved semantic categorical organization in mild cognitive impairment: A network analysis of verbal fluency. Neuropsychologia 157:107875. doi: 10.1016/j.neuropsychologia.2021.107875, PMID: 33930387

[ref23] Pakhomov S. V. S. Hemmy L. S. (2014). A computational linguistic measure of clustering behavior on semantic verbal fluency task predicts risk of future dementia in the nun study. Cortex 55, 97–106. doi: 10.1016/j.cortex.2013.05.009, PMID: 23845236 PMC4402214

[ref24] Pennington J. Socher R. Manning C. D. (2014). GloVe: Global vectors for word representation. Empirical methods in natural language processing (emnlp), 31, 682–687

[ref25] Raoux N. Amieva H. Le Goff M. Auriacombe S. Carcaillon L. Letenneur L.. (2008). Clustering and switching processes in semantic verbal fluency in the course of alzheimer’s disease subjects: Results from the PAQUID Longitudinal Study. Cortex 44, 1188–1196. doi: 10.1016/j.cortex.2007.08.019, PMID: 18761132

[ref26] Rofes A. de Aguiar V. Ficek B. Wendt H. Webster K. Tsapkini K. (2019). The Role of Word Properties in Performance on Fluency Tasks in People with Primary Progressive Aphasia. J. Alzheimers Dis. 68, 1521–1534. doi: 10.3233/JAD-180990, PMID: , 30909222 PMC6548439

[ref27] Rohrer D. Wixted J. T. Salmon D. P. Butters N. (1995). Retrieval From Semantic Memory and Its Implications for Alzheimer’s Disease. J. Exp. Psychol. Learn. Mem. Cogn. 21, 1127–1139. doi: 10.1037/0278-7393.21.5.1127 8744958

[ref28] RStudio Team. (2022). RStudio: Integrated Development for R. Available at: http://www.rstudio.com/

[ref29] Sailor K. M. Zimmerman M. E. Sanders A. E. (2011). Differential impacts of age of acquisition on letter and semantic fluency in Alzheimer’s disease patients and healthy older adults. Q. J. Exp. Psychol. 64, 2383–2391. doi: 10.1080/17470218.2011.596660, PMID: 21851152 PMC3671596

[ref30] Sauzéon H. Raboutet C. Rodrigues J. Langevin S. Schelstraete M. A. Feyereisen P.. (2011). Verbal Knowledge as a Compensation Determinant of Adult Age Differences in Verbal Fluency Tasks over Time. J. Adult Dev. 18, 144–154. doi: 10.1007/s10804-010-9107-6

[ref31] Shao Z. Janse E. Visser K. Meyer A. S. (2014). What do verbal fluency tasks measure? Predictors of verbal fluency performance in older adults. Front. Psychol. 5, 1–10. doi: 10.3389/fpsyg.2014.00772, PMID: 25101034 PMC4106453

[ref32] Troyer A. K. Moscovitch M. Winocur G. (1997). Clustering and switching as two components of verbal fluency: Evidence from younger and older healthy adults. Neuropsychology, 11, 138–556 146, doi: 10.1037/0894-4105.11.1.138, PMID: 9055277

[ref33] Troyer A. K. Moscovitch M. Winocur G. Leach L. Freedman M. (1998). Clustering and switching on verbal fluency tests in Alzheimer's and Parkinson's disease. J. Int. Neuropsychol. Soc. 4, 137–143. doi: 10.1017/s1355617798001374, PMID: , 9529823

[ref34] van Beilen M. Pijnenborg M. van Zomeren E. H. van den Bosch R. J. Withaar F. K. Bouma A. (2004). What is measured by verbal fluency tests in schizophrenia? Schizophr. Res. 69, 267–276. doi: 10.1016/j.schres.2003.09.007 15469198

[ref35] Van Den Berg E. Jiskoot L. C. Grosveld M. J. H. Van Swieten J. C. Papma J. M. (2017). Qualitative Assessment of Verbal Fluency Performance in Frontotemporal Dementia. Dement. Geriatr. Cogn. Disord. 44, 35–44. doi: 10.1159/000477538, PMID: 28624827

[ref36] Vonk J. M. J. Rizvi B. Lao P. J. Budge M. Manly J. J. Mayeux R.. (2019). Letter and Category Fluency Performance Correlates with Distinct Patterns of Cortical Thickness in Older Adults. Cereb. Cortex 29, 2694–2700. doi: 10.1093/cercor/bhy138, PMID: , 29893804 PMC6519688

[ref9001] Zehr J. Schwarz F. (2018). PennController for Internet Based Experiments (IBEX). doi: 10.17605/OSF.IO/MD832

